# Deep-learning–based optical coherence tomography reconstruction for high-speed and contrast morphology and vasculature imaging

**DOI:** 10.1117/1.JBO.31.2.025001

**Published:** 2026-02-04

**Authors:** Yudan Chen, Shuo Chen, Jun Song, Da Ma, Mirza Faisal Beg, Zaid Mammo, Myeong Jin Ju

**Affiliations:** a University of British Columbia, School of Biomedical Engineering, Faculty of Medicine and Applied Science, Vancouver, British Columbia, Canada; b Simon Fraser University, School of Engineering Science, Burnaby, British Columbia, Canada; c Wake Forest University, School of Medicine, Winston-Salem, North Carolina, United States; d University of British Columbia, Department of Ophthalmology and Visual Sciences, Vancouver, British Columbia, Canada

**Keywords:** optical coherence tomography, angiography, ophthalmology, deep neural network, balanced detection, image reconstruction

## Abstract

**Significance:**

Spectral-domain optical coherence tomography (SD-OCT) has been widely used in clinical ophthalmic imaging for high spatial resolution and phase stability. The implementation of multiple spectrometers could help resolve the challenges of SD-OCT, including limited imaging speed and sensitivity. However, these two improvements cannot be achieved concurrently.

**Aim:**

We propose a deep-learning–based approach to enhance both imaging speed and sensitivity of SD-OCT systems using a modified U-Net architecture.

**Approach:**

This network adopts a visual state space model to synthesize the high signal-to-noise ratio (SNR) OCT and OCTA from high-speed acquisitions, which bypasses the hardware restriction. The model performance is evaluated both qualitatively and quantitatively using the multiscale structural similarity index measure (MS-SSIM) and contrast-to-noise ratio (CNR).

**Results:**

The results demonstrate effective performance in high-SNR OCT/OCTA reconstruction, providing better contrast between the retinal layers and improved delineation of layer boundaries. Fine structures in both the inner and outer retina, such as microcapillaries and choroid, are successfully restored.

**Conclusions:**

We proposed an effective approach to improve the OCT image quality while maintaining the high acquisition speed using the DNN-based architecture, enabling simultaneous benefits of high imaging speed and enhanced sensitivity.

## 1 Introduction

Spectral-domain optical coherence tomography (SD-OCT) has proven invaluable in various applications, particularly in ophthalmic imaging, where it offers advanced spatial resolution and phase stability. Despite its advantages, key limitations of SD-OCT are its restricted image acquisition speed and sensitivity. In addition, the inherent sensitivity roll-off governed by the optical components of spectrometers significantly limits the performance of SD-OCT systems. Various strategies have been proposed to overcome these speed limitations, with one practical solution being the use of multiple spectrometers for detection.^1^^,^^2^ Recently, our group developed a dual-spectrometer system,^3^ which can achieve 500-kHz depth scan (A-scan) rates in high-speed mode. Alternatively, the system can operate in balanced detection mode (DBD mode), offering enhanced sensitivity and signal-to-noise ratio (SNR).^4^ To mitigate the challenge of precise alignment raised by implementing an additional spectrometer, computational methods have demonstrated effectiveness in compensating for spectral mismatches, including numerical calibration,^3^ image-based cross-calibration,^5^ and adaptive balance algorithm.^6^ Although these approaches can improve the performance of dual-spectrometer systems, they remain incapable of simultaneously taking advantage of both modes. This limitation originates from the fundamental differences in spectrometer operation for each mode: high-speed mode requires the alternating operation of line-scan cameras, whereas DBD mode requires synchronized operation.

These hardware constraints highlight the need for alternative solutions to improve the image quality. Recent advancements in machine learning motivate the implementation of deep neural networks (DNNs) in visual perception tasks. In the field of image reconstruction, convolutional neural networks (CNNs) have been a cornerstone since the introduction of super-resolution CNNs.^7^^,^^8^ Recent studies incorporated the residual blocks^9^^,^^10^ or transformers^11^ to improve the performance in feature extraction and pattern recognition.^10^ Generative adversarial networks (GANs) are another widely implemented model that have shown promising results in image synthesis.^12^^,^^13^ However, these models either lack the ability in fine texture recovery and capture long-range dependencies^14^ or are challenged by training stability due to the inherent nature of the optimization process between the generator and discriminator.^15^

In this letter, we introduce a DNN-based approach to improve the SNR of OCT images acquired with the high-speed mode of the dual spectrometer system, utilizing a modified U-Net architecture with VMamba^14^ as the backbone, referred to as VM-UNet. The significance of the proposed architecture will be evaluated, and the performance will be demonstrated, which ultimately leads to exploiting the advantages of both high-speed and DBD modes of the dual-spectrometer system simultaneously. The efficacy of the proposed model will be assessed by a comprehensive comparison across the images acquired by both modes and model outputs.

## 2 Methods

### 2.1 Backbone Selection

VMamba is a recently introduced architecture based on a state space model (SSM), which has been demonstrated to have promising performance across multiple visual tasks.^14^ By incorporating the 2D Selective Scan (SS2D), VMamba captures the nonsequential and spatial information in 2D images while maintaining the ability of SSMs in handling the long-range dependencies with low computational overhead. These characteristics highlight the potential of VMamba for OCT image denoising and reconstruction, where both global context and spatial dependencies of retinal structures are significant.

### 2.2 Dataset Preparation

The dataset for training and validation was acquired with DBD mode using our previously developed dual-spectrometer system, whose spectrometers were numerically calibrated.^3^ A total of 25 acquisitions were obtained from healthy adult volunteers (n=8). All imaging procedures were approved by the research ethics board at the University of British Columbia (H19-03110) and conducted in accordance with the Declaration of Helsinki. The interference signals of each spectrometer were processed with conventional OCT processing to form the network input, referred to as OCT-S1 and OCT-S2, respectively. Each volume represents a 3D retinal image that covers a field of view of 4  mm×4  mm centered at the fovea. Along the fast transverse (X) direction, each B-scan comprises 500 A-scans, with each A-scan sampled by 550 points along the axial (*Z*) direction. In the slow transverse (*Y*) direction, a total of 1500 B-scans are obtained, with each *Y* position scanned three consecutive times (BM-scans) to generate OCTA images. The acquisition time per volume is 3.3 s under the A-scan rate of 250 kHz in DBD mode. For each B-scan, every other A-scan was alternated between OCT–S1 and OCT–S2 to simulate the high-speed acquisition, yielding a total of 50 volumes with 75,000 OCT B-scans. The balanced detection OCT and OCTA derived from OCT-S1 and OCT-S2 were processed to form the ground truth. Instead of converting to image files where pixel values need to be normalized to integers in the range between 0 and 255, the VM-UNet utilizes the OCT data in the form of floating-point values to minimize information loss. The model robustness and generalization were evaluated using the dataset acquired under high-speed mode. Due to the nature of high-speed mode that operates each spectrometer alternatively to maximize the duty cycle of the camera,^3^ balanced detection OCT and OCTA volumes were not generated for comparison.

### 2.3 Architecture Design

**Fig. 1:**
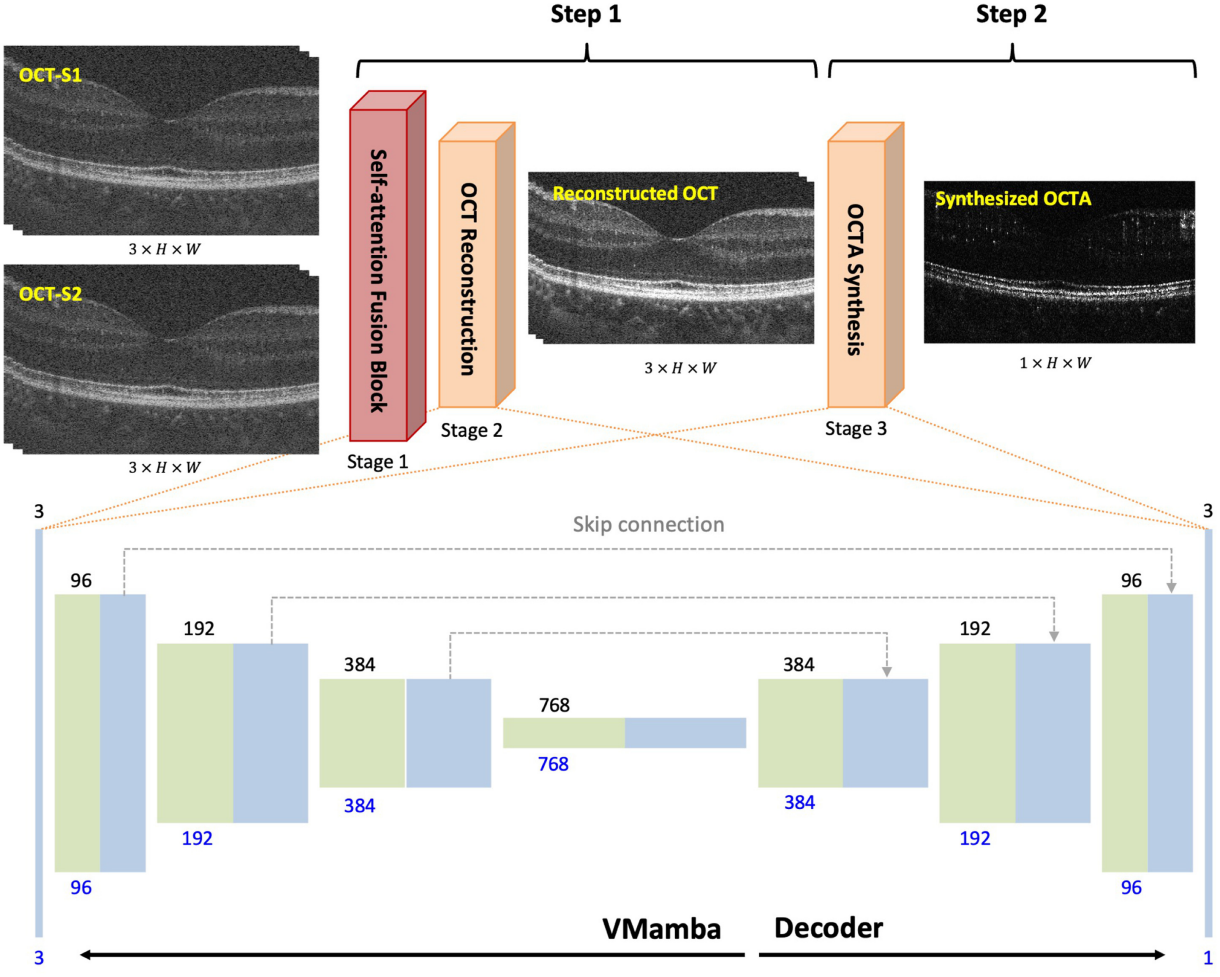
Architecture of the dual-step VM-UNet for high-SNR OCT and OCTA reconstruction.

The architecture of dual-step VM-UNet is illustrated in Fig. 1. The proposed network is designed to reconstruct OCT and OCTA sequentially in two steps. The first step focuses on generating high-SNR OCT. The BM-sets of both spectrometers serve as the inputs of the network. The paired OCT BM-set was first concatenated and passed to the self-attention fusion block (stage 1), which captures and combines the complementary features across the two spectrometers. The resulting three-channel feature map was subsequently passed to stage 2 for OCT reconstruction. This stage utilizes a framework that follows the design of U-Net using the VMamba as the encoder. The output of this step is a three-channel high-SNR OCT image, with each channel representing a BM-scan. The second step takes the resulting OCT as the input to synthesize OCTA. In stage 3, a similar UNet-like framework was applied, differing only in the number of output channels in the final decoder block. All stages are trained end-to-end with a unified validation loss, enabling mutual information learning across OCT and OCTA. Each block of the UNet-like framework in Fig. 1 represents multi-channel feature maps, with the channel numbers annotated at the top (stage 2) and bottom (stage 3). The training process was performed on two NVIDIA Quadro RTX 8000 GPUs allocated through the Cedar cluster of the Digital Research Alliance of Canada. The model was trained and validated on a total of 66,000 OCT B-scan images from 44 volumes through five-fold cross-validation, with 9000 B-scans from six volumes reserved for testing. The training-to-validation was split at the volume level with a split ratio of 4:1. To mitigate the effect of small cohort size, data augmentation was applied, including random horizontal flipping, random rotations, Gaussian noise addition, and isotropic scaling by a factor uniformly sampled from 0.9 to 1.1. The model hyperparameters were optimized using the AdamW optimizer with an initial learning rate of 0.0001. The learning rate was dynamically adjusted using the ReduceLROnPlateau scheduler to prevent training stagnation and was reduced by half when the validation peak SNR (PSNR) showed no improvement for two consecutive epochs. The early stopping was triggered when the validation PSNR plateaued with a patience of three epochs, ensuring optimal generalization while minimizing overfitting. The overall training objective combined three complementary loss functions: Huber, structural similarity index measure (SSIM), and perceptual losses. At stage 2, these losses were computed with respect to the ground-truth OCT image, whereas at stage 3, they were evaluated against the ground-truth OCTA image.

## 3 Results

### 3.1 Testing Set Assessment

**Fig. 2:**
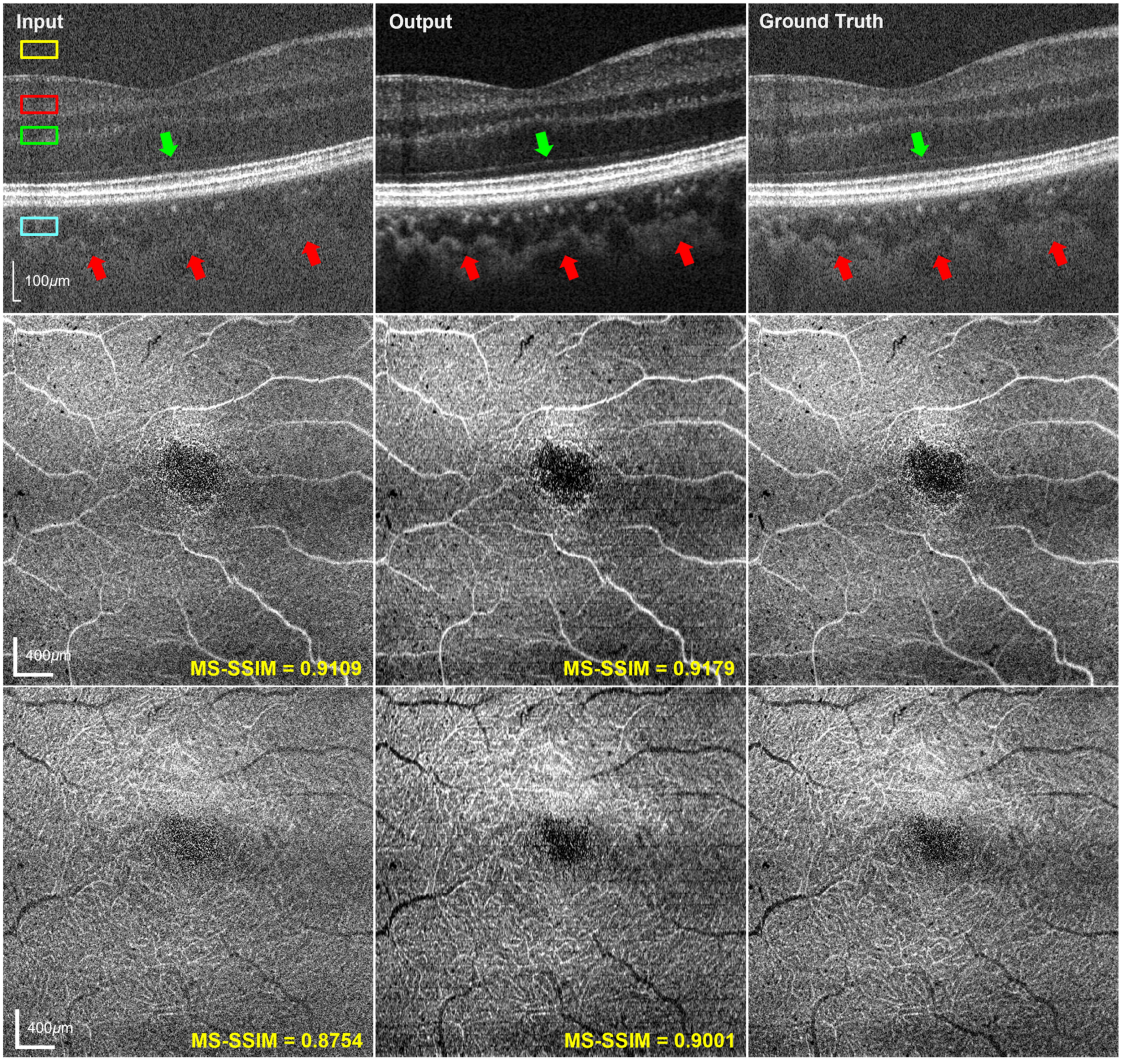
Comparison of OCT images across input, output, and ground truth in cross-sectional and *en face* view, including B-scans (top), SVP (middle), and DVP (bottom). Representative anatomical structures are indicated by arrows in B-scans to show contrast improvement, including the IS/OS junction (green) and the choroid (red). Four ROIs are selected from the background area (yellow), SVP (red), DVP (green), and choroid (blue) for CNR calculation.

The reconstructed OCT of the testing data is shown in Fig. 2. The output B-scan demonstrates significant noise suppression, providing improved contrast between the retinal layers and better delineation of the layer boundaries. As indicated by the arrows, the photoreceptor inner segment/outer segment (IS/OS) junction (green) and choroid structures (red) are clearly visible in the output. Image quality improvement is quantified with contrast-to-noise ratio (CNR) for a measurement of contrast between the tissue and the background. Representative regions of interest (ROIs) are manually selected from the superficial vascular plexus (SVP), deep vascular plexus (DVP), and choroid. The SVP covers layers extending from the lower boundary of the retinal nerve fiber layer to the lower boundary of the inner plexiform layer (IPL), whereas the DVP spans from IPL to the lower boundary of the outer plexiform layer. Each ROI was defined as a 20×40  pixel rectangle, as indicated by the colored boxes in Fig. 2, where yellow is the background while others are the layers with retinal capillaries, which are selected from identical A-scans to compare image quality before and after reconstruction along the depth. As summarized in Table 1, the output achieved CNR values more than 3 times higher than the input across all regions, demonstrating substantial enhancement in image quality. To have a better visualization of the vascular networks, projection images of the SVP and DVP were presented. The large vessels in the SVP show enhanced contrast in both output and ground truth, whereas the thin microcapillaries in the DVP demonstrate more pronounced improvement in vessel visibility and connectivity. To quantitatively assess the structural information restoration, the multiscale SSIM (MS-SSIM) was computed for *en face* OCT images on both input and output using the ground truth as the reference. As shown in Fig. 2, an increase in the MS-SSIM score particularly in DVP from 0.8754 to 0.9001 highlights the promising performance of the model in reconstructing fine structures in OCT.

**Table 1:** Image quality measurements of Fig. 2.

Metrics	CNR		
	SVP	DVP	Choroid
Input	3.060 ± 0.235	0.733 ± 0.413	3.319 ± 0.264
Output	13.940 ± 0.215	10.794 ± 0.349	12.561 ± 0.769

**Fig. 3:**
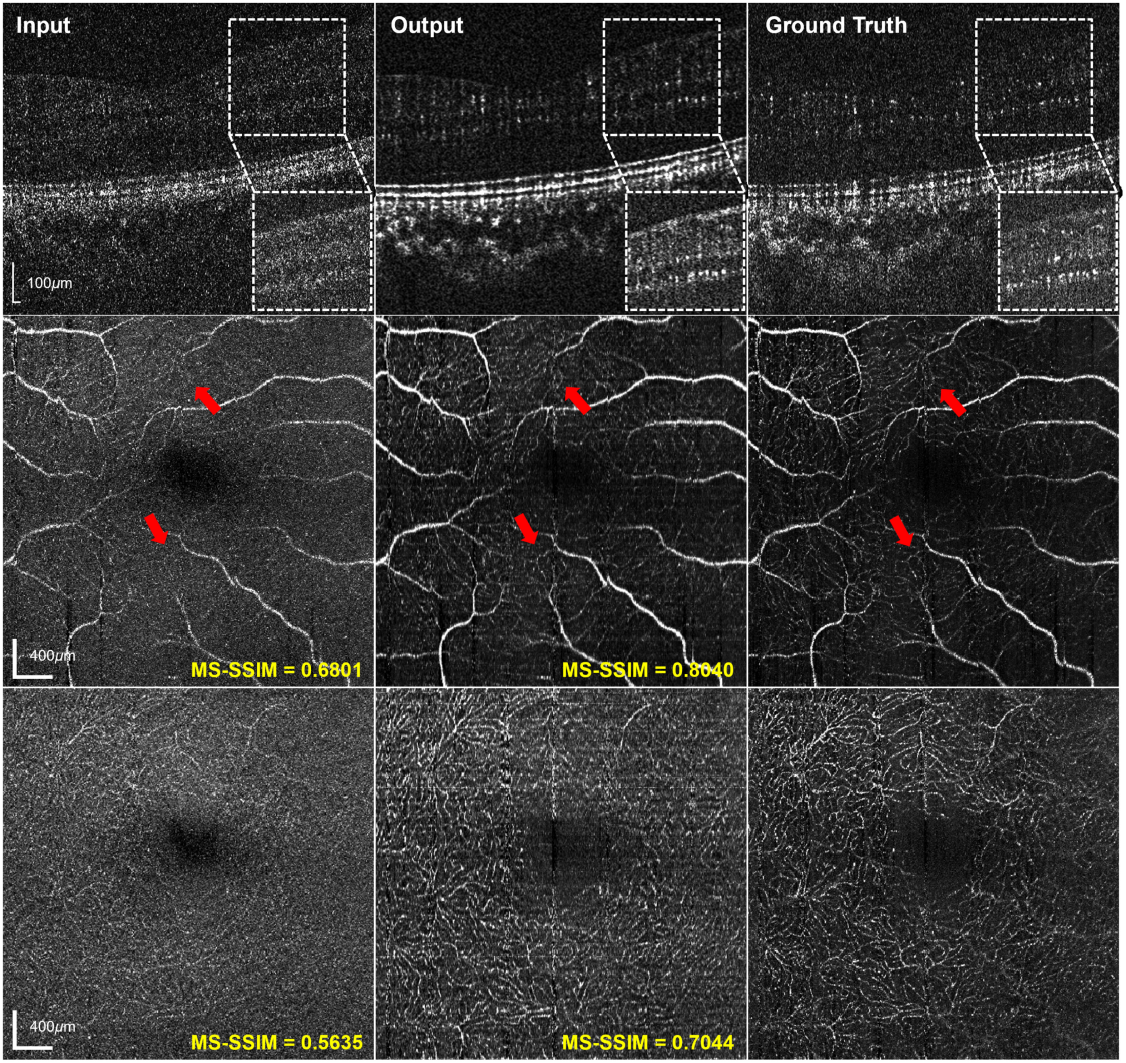
Comparison of OCTA images across the input, output, and ground truth in cross-sectional and *en face* view, including B-scans (top), SVP (middle), and DVP (bottom). ROIs within the inner retina are indicated by white dashed boxes in the B-scans, and the corresponding magnified views are shown at the bottom right to highlight contrast improvement after reconstruction. Representative vessel structures are indicated by the red arrow in the *en face* SVP images.

The synthesized OCTA signals illustrated in Fig. 3 exhibit enhanced contrast relative to surrounding fixed tissues. In the representative B-scans, ROIs within the inner retina are indicated by white dashed boxes, and the corresponding subimages show magnified views of these regions to highlight the contrast improvement, where the image contrast is less influenced by the hyper-reflective region around the retinal pigment epithelium. In the *en face* OCTA, the thick vessels in the SVP are more distinctly distinguishable from the background, accompanied by improved depiction of the surrounding branching capillary structures (red arrows). Moreover, the output DVP reveals better reconstruction of vascular morphology, providing sharper delineation and clearer visualization of microcapillaries. The MS-SSIM score further demonstrated the effectiveness of the model in synthesizing high-SNR OCTA signals, with significant improvements observed in both SVP and DVP.

### 3.2 Performance Verification

**Fig. 4:**
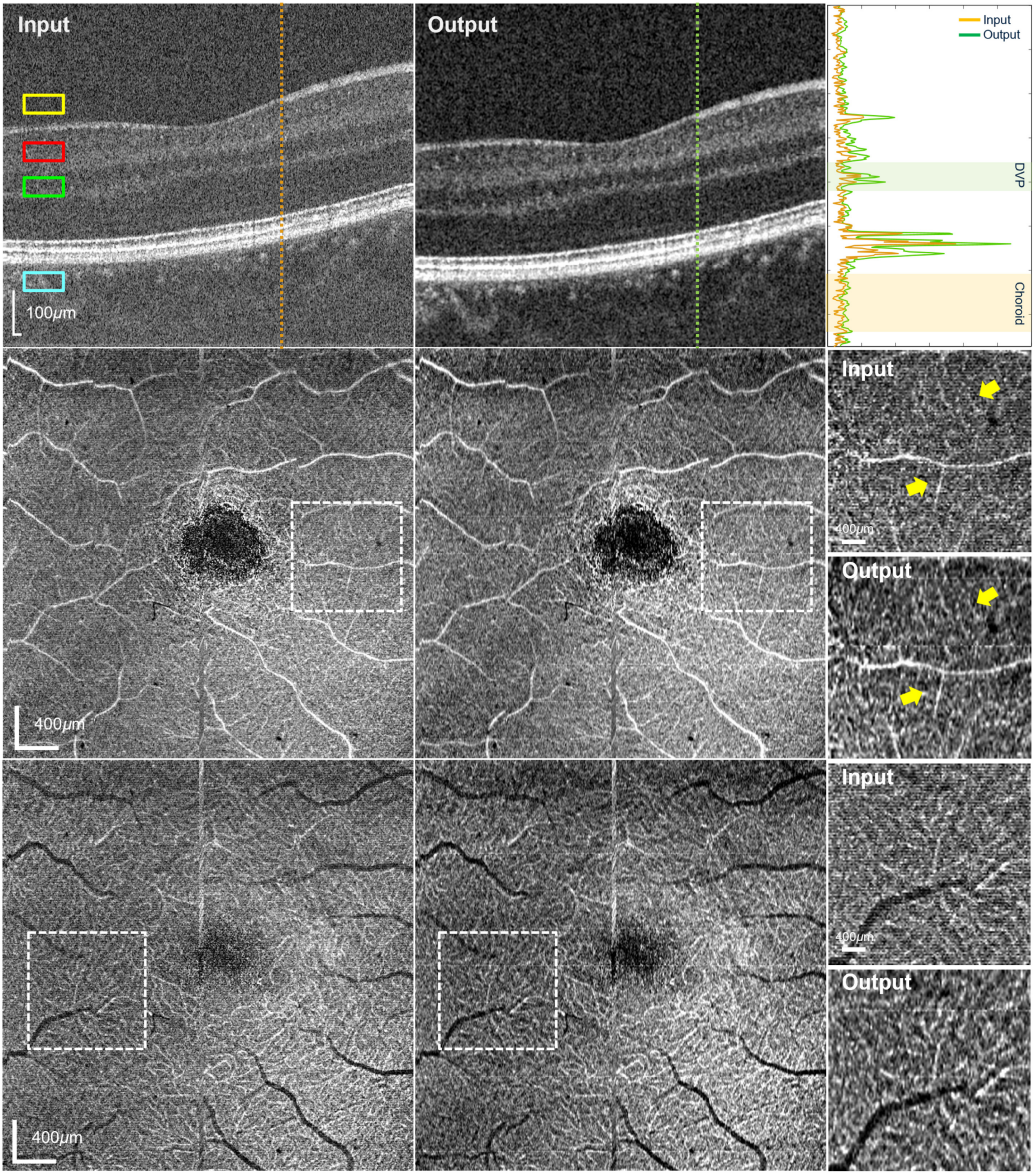
Comparison of OCT images from the high-speed dataset between input and output in cross-sectional and *en face* view, including B-scans (top), SVP (middle), and DVP (bottom). A representative A-scan from the input (orange) and output (green) B-scan is selected to generate the corresponding A-scan intensity profile. ROIs in the *en face* OCT images are indicated by white dashed boxes, and the corresponding magnified views are shown on the right to highlight vessel signal enhancement after reconstruction.

**Fig. 5:**
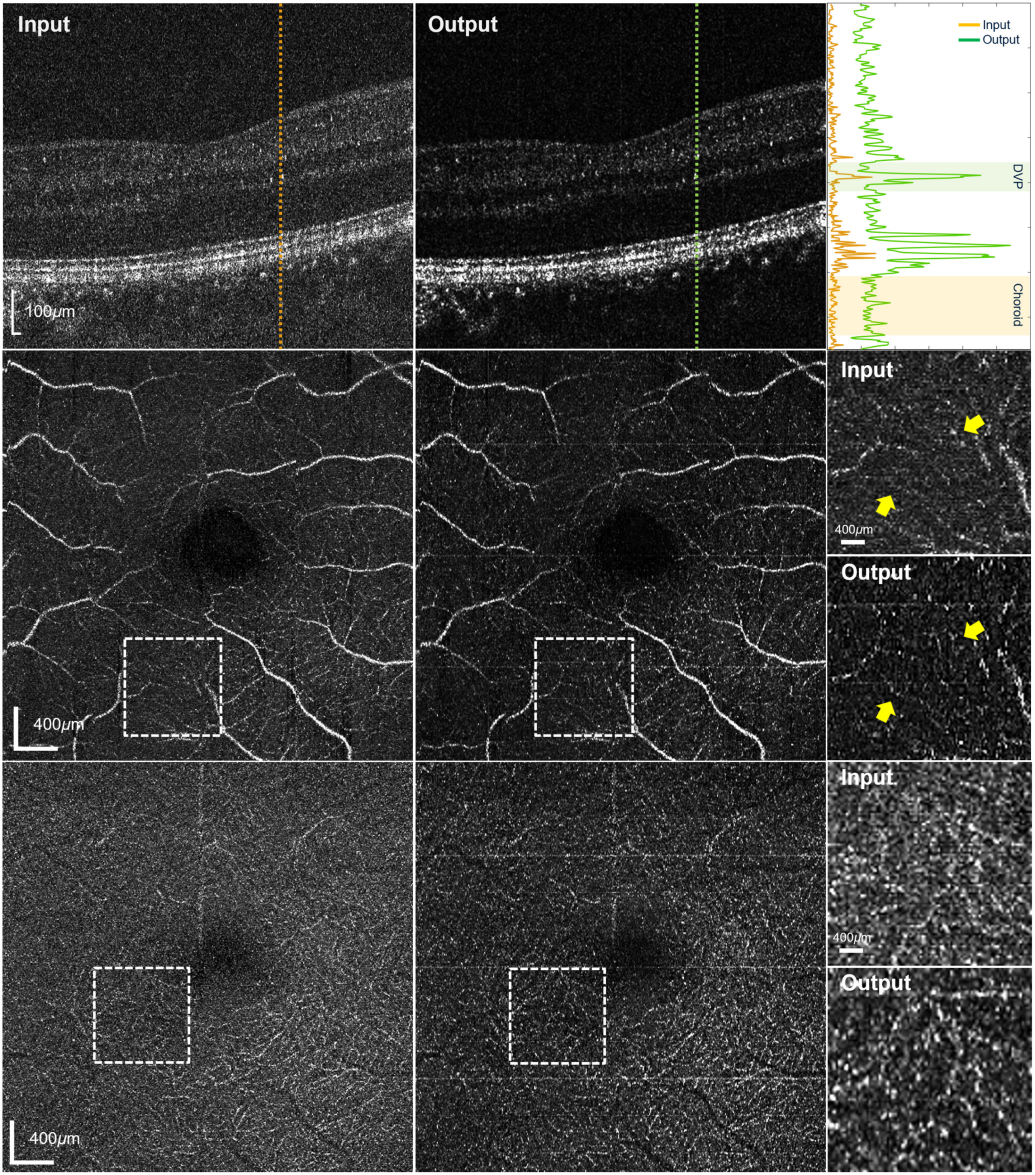
Comparison of OCTA images from high-speed dataset between input and output in cross-sectional and *en face* view, including B-scans (top), SVP (middle), and DVP (bottom). A representative A-scan from the input (orange) and output (green) B-scan is selected to generate the corresponding A-scan intensity profile. ROIs in the *en face* OCTA images are indicated by white dashed boxes, and the corresponding magnified views are shown on the right to highlight vessel signal enhancement after reconstruction.

The model performance on high-SNR OCT/OCTA reconstruction from high-speed acquisitions is presented in Figs. 4 and 5. The output OCT B-scan shows significant noise suppression, with increased signal intensity of the structures between retinal layers as demonstrated in both A-scan profiles. Table 2 summarizes the CNR analysis, which showed approximately triple improvement over the input, consistent with the testing data. The reconstruction of capillary networks is further illustrated in *en face* visualizations. As indicated by the yellow arrows in the zoomed-in views, capillary networks surrounding the thick vessels show improved connectivity and visibility in both OCT and OCTA in the SVP, whereas the microcapillaries in the DVP display better contrast and delineation.

**Table 2:** Image quality measurements of Fig. 4.

Metrics	CNR		
	SVP	DVP	Choroid
Input	5.836 ± 0.176	1.088 ± 0.388	5.098 ± 0.501
Output	14.958 ± 0.175	9.120 ± 0.453	12.466 ± 0.670

## 4 Discussion

This study presents an effective approach that incorporates the advantages of both modes of the dual-spectrometer system simultaneously using a DNN model. Unlike traditional convolutional or transformer-based networks,^16^^,^^17^ VMamba utilizes linear recurrence mechanisms, enabling it to process sequences with high efficiency while preserving global contextual information.^14^ Its ability to model complex spatial information with low computational overhead enables effective reconstruction of high-quality images from degraded inputs. This makes VMamba a viable solution for medical image reconstruction, such as OCT and OCTA, where spatial dependencies and fine structural details are crucial.

The application of the proposed approach for enhancing sensitivity could provide a significant benefit for conventional SD-OCT systems, which typically suffer from limited penetration depth owing to shorter wavelengths of the laser sources. In addition, the inherent sensitivity roll-off of SD-OCT systems can lead to the loss in signal intensity in both OCT and OCTA,^18^ especially in DVP and choroid, which can lead to diagnosis difficulties as layer thickness and capillary density have been correlated with early pathological changes in various retinal diseases.^19^

Although previous studies demonstrated promising denoising performance using machine learning, over-smoothing and blurriness have been commonly reported,^20^^,^^21^ which potentially leads to the loss of information on capillary details. The proposed VM-UNet is able to reconstruct high-SNR OCT images from high-speed mode acquisitions and also generate high-SNR OCTA images inferred from three repeated OCT scans. By utilizing the OCT floating-point amplitude data, the information represented by the originally acquired values is preserved to maintain the data fidelity, which is crucial when further generating additional contrast, such as OCTA.

As shown in Figs. 3 and 5, the reconstructed OCTA of high-speed acquisitions shows improved contrast of vessel signals, providing better vascular texture details with high imaging speed. In particular, the reconstructed *en face* OCTA images (Fig. 3) show increased MS-SSIM values compared with the input but are generally lower than those of *en face* OCT (Fig. 2). This phenomenon is potentially due to the difference in image characteristics of the two contrasts. Because OCTA is the motion contrast derived from repeated OCT acquisitions that shows the blood vessel structures, its sparse nature and high sensitivity to pixel-level intensity variations limit the achievable MS-SSIM despite visible improvements after reconstruction. Nevertheless, the enhanced visualization of vascular structure is clinically significant for the diagnosis of retinal vascular disorders such as diabetic retinopathy^22^ and retinal vein occlusion.^23^

High acquisition speed is critical in clinical applications for minimizing motion artifacts, which cause significant structural distortions that hinder precise visualization of morphologies and capillaries in retinal images. It also enables the acquisition of more BM-scans, which can be further leveraged for advanced analyses such as variable interscan time–based quantitative retinal blood flow assessment. The proposed framework is designed to improve the image acquisition time and quality simultaneously for a dual-spectrometer SD-OCT system, which reconstructs the images on a B-scan basis. Although the current evaluation was performed during postprocessing, the computational structure of the method suggests that real-time image enhancement could be achieved without a practical delay in clinical workflow.

However, this study has several limitations. First, the current training and validation dataset only includes the healthy subjects, where the reconstruction performance on pathological cases remains uncertain. Second, the advantages of using VMamba to learn spatial dependencies and fine features require further validation through benchmarking against the state-of-the-art networks, such as convolutional neural networks and vision transformers. For future studies, the application of VM-UNet with the dual-spectrometer system can be further expanded by incorporating OCT phase information, which has the potential to provide additional OCT contrasts, including the degree of polarization uniformity and the attenuation coefficient, as well as phase-resolved OCTA reconstruction.

## 5 Conclusion

In conclusion, we proposed an effective approach to reconstruct balanced detection OCT and OCTA using a DNN model to bypass the hardware restrictions. The DNN model, which employed a visual SSM to better capture both local information and global spatial dependencies in vision data, has been demonstrated to generate high-SNR images without compromising the fine details. The application of the introduced DNN-based approach to generate high-SNR images from high-speed acquisitions is expected to be significantly beneficial in both research and clinical environments.

## Data Availability

Data underlying the results presented in this paper are not publicly available at this time but may be obtained from the authors upon reasonable request.
